# Laparoscopic Intraperitoneal Onlay Mesh (IPOM) in the Treatment of Ventral Hernias: Technique Discussion Points

**DOI:** 10.7759/cureus.61199

**Published:** 2024-05-27

**Authors:** Iulian M Slavu, Florin Filipoiu, Octavian Munteanu, Raluca Tulin, Bogdan Ursuț, Iulian A Dogaru, Anca Monica Macovei Oprescu, Ileana Dima, Adrian Tulin

**Affiliations:** 1 Anatomy, Carol Davila University of Medicine and Pharmacy, Bucharest, ROU; 2 Endocrinology, Agrippa Ionescu Clinical Hospital, Bucharest, ROU; 3 General Surgery, Agrippa Ionescu Clinical Hospital, Bucharest, ROU; 4 Medicine, Carol Davila University of Medicine and Pharmacy, Bucharest, ROU; 5 Gastroenterology, Agrippa Ionescu Clinical Hospital, Bucharest, ROU

**Keywords:** ventral wall hernias, ipom plus, intraperitoneal onlay mesh (ipom), incisional ventral hernia, laparoscopic ventral hernia repair

## Abstract

Incisional ventral hernias (IVH) are a common occurrence worldwide. The resolve is fundamentally surgical. In this regard, laparoscopic treatment has become the standard. This paper aims to review intraperitoneal onlay mesh (IPOM) as a surgical solution for IVH and to explore the limitations and advantages in relation to the technique of mesh fixation, defect suture, seroma formation, and recurrence in accordance with the data published. The article is structured as a narrative review and relies on the Scale for the Assessment of Narrative Review Articles (SANRA) convention. In the analysis, we included articles published in the literature regarding the surgical treatment of ventral hernias (umbilical and incisional) through the IPOM technique. We explored data regarding the mesh fixation technique on the anterior abdominal wall (tacks or sutures), indications and limitations of defect closure, incidence of seroma formation, and recurrence rate. Laparoscopic IPOM is a better option for IVH up to 10 cm than the open technique with regard to aesthetics, length of hospital stay, and postoperative pain. There is no difference in recurrence rates. Suturing of the defect should be done to decrease seroma formation and maintain the functionality of the abdominal wall. Ideally, the suture should be done intraperitoneally or laparoscopically. Regarding pain in mesh fixation, there seems to be an increase in the short-term postoperative pain in the suture groups, but at six months, when compared to the tacks groups, there is no difference. New methods are being developed that include different types of glue but require large prospective, randomized trials if they are to be included in the guidelines.

## Introduction and background

Incisional ventral hernias (IVH) are a common occurrence worldwide. The resolve is fundamentally surgical. In this regard, laparoscopic treatment has become a standard if the resources are available. Consecutive meta-analyses published by Zhang et al. and Chelala et al. demonstrated lower recurrence (4.4-4.7%) and reduced postoperative complications compared to the open technique [1,2]. Another encompassing Cochrane review published by Sauerland et al. demonstrated a decreased incidence of wound infection and a shorter hospital stay [3]. From the arsenal of laparoscopic procedures for IVH, intraperitoneal onlay mesh standard (IPOMs; without defect closure) and intraperitoneal onlay mesh plus (IPOMp; with defect closure) stand out as relatively simple procedures that require minimum dissection and offer good postoperative results in regards to recurrence, pain, wound infection, and length of hospital stay. The International Endohernia Society recommends the procedure for IVH with defects up to 10 cm in diameter [4]. LeBlanc published the first ventral hernia repair through the IPOM technique in 1993 [5]. The procedure is relatively straightforward and involves laparoscopic dissection and reduction of the hernia sac, followed by bridging the defect with a mesh from the peritoneal side. As it was adopted on a larger scale, problems and complications began to be reported. These relate to the methods used to anchor the mesh to the abdominal wall: sutures or tacks. Sutures demonstrated higher short-term postoperative pain, while tacks have complications related to wall hematomas or even intestinal perforation due to adhesions [6]. Another disputed aspect of the procedure is associated with the fact that in IPOMs, the defect is not sutured, which leads to seroma formation, mesh bulging, and higher recurrence rates [7]. Consequently, IPOMp was envisioned to circumvent this problem, in which the defect is sutured before placing the mesh. But this again raised other questions about when and what size the defect should be sutured and through what technique. If they are sutured, more significant defects are in tension and can lead to increased postoperative pain [8].

This paper aims to review IPOM as a surgical solution for IVH and explore the limitations and soft points in relation to the technique of mesh fixation, defect suture, seroma formation, and recurrence in accordance with the published data.

## Review

Search strategy and selection criteria

The article is structured as a narrative review and relies on the Scale for the Assessment of Narrative Review Articles (SANRA) convention. In the analysis, we included articles published in the literature regarding the surgical treatment of ventral hernias (incisional) through the IPOM technique. We explored published data regarding the mesh fixation technique on the anterior abdominal wall (tacks or sutures), indications and limitations of defect closure, incidence of seroma formation, and recurrence rate. The studies were obtained from the PubMed database and the Scopus medical database. The keywords used were IPOM standard, IPOM plus, laparoscopy in ventral hernia, seroma in IPOM, recurrence in IPOM, mesh fixation in IPOM, and defect closure in IPOM. Articles not in English, commentaries, or notes were not included. A total of 40 articles were selected and included in the discussion. Two authors made the selection to ensure unbiased decisions.

Defect closure and seroma formation

In accordance with the current guidelines, IPOM is reserved for defects under 10 cm in diameter [1]. If the diameter exceeds 10 cm, the recurrence rate and postoperative pain increase, mainly due to the high tension of the knots used to close the breach [9,10]. In this situation, posterior component separation with transverse muscle release (TAR), either laparoscopic (extended totally extraperitoneal repair) or open, should be the ideal option. The operation's main objective for IVH is to redo the anatomy and integrity of the abdominal wall. In this regard, the fascial defect needs to be closed with sutures and reinforced with mesh. The first mentioned procedures of IPOM noted only a bridging method where the mesh is placed over the defect while the breach in the abdominal is left open. Thus, the procedure was termed “tension-free.” This decision presents itself with a set of problems, most related to seroma formation in the remaining unclosed cavity. The collection will protrude under the skin, similarly to the hernia. The patient can and will regard this aspect as a recurrence, although these resorb in four to six months. Regarding the incidence, the data in the literature is contradictory. Seroma seems to occur in 30% of IPOMs [11]. Clapp et al. mentioned better outcomes for IPOMp in a retrospective study of 176 patients. In the IPOMs group, the incidence of seroma formation was 27%, while in the IPOMp, it was 5% (p=0.02) [12]. Then again, Zeichen et al., in a much smaller group consisting of 110 patients, observed that in the IPOMp group, of the eight patients with postoperative complications, four had seromas. In percentages, it seems high (50% of all the postoperative complications), but they did not reach statistical significance [13]. Pizza et al., in a prospective multicenter trial with a follow-up of 36 months where they compared IPOMs vs. IPOMp, did not observe any statistically significant difference regarding wound events and put the discordant results in the literature on the type of mesh used, type of closure, or suture technique [14]. Besides seroma formation, a proper abdominal wall needs to provide trunk movement, protection of intraperitoneal organs, and aid in physiologic functions (defecating, urinating, vomiting, and breathing). As a consequence, it needs to be homogenous and without any structural defects. These aspects lead to the logical assumption that the defect suture is essential for preserving these functions. Again, there is a lack of objective data with regard to how defect closure impacts physiologic functions. Den Hertog et al. demonstrated that the trunk flexor muscles' isokinetic strength is diminished after IVH surgery. In this regard, they observed that suturing the defect resulted in greater strength in flexing movements, especially in isokinetic ones, compared to untutored defects [15]. They pointed out that the reduced strength of the trunk flexor muscles after surgery for IVH without sutures can lead to chronic back pain, as these muscles will have to compensate for the reduced strength of the anterior muscles. The study group consisted of only 30 patients. There is an astounding lack of data regarding the functionality of the anterior abdominal wall after hernia treatment. However, this is one of the main reasons why a patient has surgery [16]. Regarding the functionality of the anterior abdominal wall (defecating, urinating, vomiting, and breathing), Clapp et al. observed better functional status after IPOMp than after IPOMs (79 vs. 71, p-0.002). The study group consisted of 176 patients, and the results were statistically significant [12]. The authors inferred the option not to close the defect from the pain generated by the tension suture [12]. Suwa et al., in a review where they analyzed over 14 studies regarding IPOMs vs. IPOMp, noted that there is currently no standard for how to evaluate chronic pain after surgery for IVH as it is in inguinal hernias, where chronic pain is defined as persistence over six months [17]. They identified only one study that measured this variable (Clapp et al.) and confirmed it was lower in the IPOMp (9%) than in the IPOMs (18%), but there wasn’t a statistical difference [12]. Another argument in favor of closing the defect is provided by Bittner et al. and Berney et al. They point out that the mesh will be in direct contact with the abdominal wall after closure, facilitating better integration and scar formation [18,19].

The options for closing the defect vary in response to multiple variables. The sutures can be done intracorporeally or extracorporeally and can be interrupted or run [16]. Orenstein et al. proposed a novel and original technique called the “shoelacing” procedure with multiple extracorporeal stab wounds on the side of the defect to pass the thread. The knots will be placed extracorporeally in the subcutaneous tissue. This decision can lead to postoperative granulomas and generate a whole range of esthetic problems [20,21]. To overcome this problem, Jani sutured the defect intracorporeally in a large prospective study on 278 patients spread over a 10-year period and reported no complications or limitations [22]. Suppose multiple minor defects exist in the anterior abdominal wall with a “Swiss cheese” aspect. In that case, the mesh can be placed without sutures, as mentioned by Clapp et al., Tatsuya et al., and Kalpesh et al., without significant postoperative consequences, but this should be the exception, not the norm [12,22,23]. If the surgeon has the necessary skills and the defects do not average more than 3 or 4, they can be sutured individually before placing the mesh, as mentioned by Grapotte et al. [24].

Recurrence

The most important endpoint of every surgical intervention for IVH is recurrence. Although laparoscopy has already been known to have advantages, it seems that in terms of recurrence, there is no difference between open IPOM and laparoscopic IPOM. Loh et al. observed no recurrence difference between open IPOM and laparoscopic IPOM (p=0.213) when they compared the patients on a lot of 100 individuals over a two-year period [24]. Kockerling et al., in a large nationwide retrospective study published in 2019, which contained almost 10.000 patients, also mentioned no recurrence rate difference between open or laparoscopic IPOM for IVH [25]. Then again, between IPOMs and IPOMp, differences seem to exist in the recurrence rate [26,27]. In this regard, Suwa et al. did a retrospective analysis of almost 2,000 patients across 15 studies. They observed that in IPOMs, the recurrence range was 0-7.7% over a postoperative monitoring period of 10-50 months [17]. Four studies mentioned lower recurrence rates for IPOMp than IPOMs (Table 1) [12,13,26,28].

**Table 1:** Recurrence at 24 months for IPOMs vs. IPOMp IPOMs: intraperitoneal onlay mesh standard, IPOMp: intraperitoneal onlay mesh plus

	Rate of recurrence at 24 months in percentage	
Study	IPOMs	IPOMp
Clapp et al. [12]	16.7	0
Zeichen et al. [13]	15.1	5.7
Banerjee et al. [26]	4.8	3.0
Toffolo Pasquini et al. [28]	9.8	15

A more recent study published in 2023 by Taşdelen et al. observed that fascia closure in IPOMp for small to medium hernias does not decrease the recurrence rate at 73 and 51 months, respectively (p=0.196) [29]. The study group consisted of 82 patients. Additionally, in 2022, Ali et al. observed 213 patients. They discovered no difference between the recurrence rate of IPOMp and IPOMs (three recurrence cases out of 98 patients vs. three recurrence cases out of 94) over a period of 36 months [18]. This large variability in recurrence rates between study groups and procedures is most probably related to defect size and the lack of homogeneity regarding BMI and smoking status. These external variables can influence the recurrence rate, irrespective of the surgical procedure.

Mesh size and fixation

**Figure 1:**
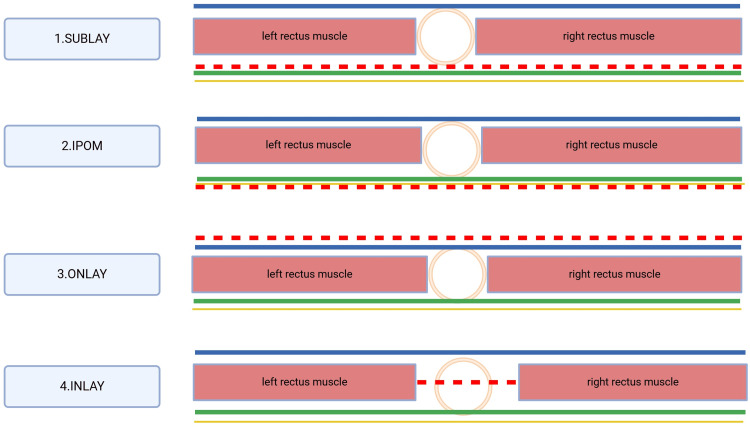
Coronal view through the rectus sheath at the level of the umbilicus, which demonstrates the different locations of the mesh placement in relation to the rectus muscle Sublay: posterior to the rectus muscle but anterior to the parietal peritoneum. IPOM: the mesh is placed posterior to the rectus muscle and posterior to the parietal peritoneum. Onlay: the mesh is placed anterior to the rectus muscle. Inlay: the mesh is placed between the two rectus muscles Blue line: anterior rectus sheath, green line: posterior rectus sheath and peritoneum, red dotted line: mesh, yellow line: peritoneum IPOM: intraperitoneal onlay mesh Image Credit: Author, original work generated at Biorender.com

The space with respect to the rectus muscles where the mesh is placed is central to the surgical success as it stabilizes the abdominal wall, offers the matrix onto which a strong scar develops, and relieves the mechanical stress on the breach [30,31]. The mesh can be placed as demonstrated in Figure 1.

In IPOM, after the mesh is introduced in the abdomen and placed on the anterior abdominal, it needs to be fixed in position. This step is critical to the outcome, and two options exist: sutures or tacks. There is a lack of high-quality prospective randomized studies that compare these two options. A prospective randomized study was done by Beldi et al., in which they compared the shrinkage and postoperative pain rate of these two fixation methods [32]. The study group was relatively small and consisted of 40 patients, but they were available for follow-up for up to six months. They observed statistical significance related to higher rates of pain in the suture group during the immediate postoperative period (p=0.008) but no difference at six months. This variability in time was attributed to entrapment, direct nerve lesions, and local inflammation. These complications lessened at six months due to complete tissue necrosis through ischemia or decreased local inflammation. One interesting aspect observed was that in the tacks subgroup, there was increased shrinkage of the mesh when compared to the suture group. This can lead to the assumption that the mesh should be increased by at least 1 cm when one uses tacks. In the tacks subgroup, the main mesh area decreased by 12% vs. 2.9% in the suture group at six months (p=0.061). Another prospective and randomized study was published by Langenbach et al., in which they measured only postoperative pain through the standard Carolinas Comfort Scale (CCS) scale [33]. They had a follow-up of 12 months and 48 patients, divided into two subgroups. There was no postoperative statistical difference in pain (although it was higher in percentages for the suture group), with an index on the CCS of 16.9 for tacks and 19.6 for sutures (p=0.20). After six months and 12 months, there was no difference. They concluded that there is no advantage to tacks over sutures regarding postoperative pain. Tacks only increased the cost. There was also no difference in the secondary endpoints such as life quality, complication rate, length of admission, or inability to work. In rare situations, as reported by Haltmeier et al. and Giuffrida et al., the tacks can cause intestinal fistulas due to adherences and erosions to the small bowel as they are exposed to the contents of the abdominal cavity [34,35]. In an extensive review published by Mathes et al., in which they included 10 trials with a total of 787 participants regarding the postoperative impact of pain produced by different fixation methods in IPOM, they concluded that the difference between groups (tacks or sutures) in the early postoperative period is negligible [36]. Glue can be used as an alternative to tacks or sutures, as it is less traumatic. Fibrin glue does not work on the intact peritoneum, as Eriksen et al. and Schug-Pass et al. demonstrated on experimental porcine models [37,38]. The peritoneum has fibrinolytic activity, which inactivates the fibrin glue [37,38]. Wilson published a more recent study on 138 patients with a follow-up of 40 months in which they used a new technology called Liquiband Fix8™ [39]. The study design did not incorporate a control group. Only two patients (1.5%) developed chronic pain that resolved in 12 months, seroma in 1%, and recurrence in 2%. The hernias had a median width of 5 cm (range: 1-9 cm) and a median length of 10 cm (range: 3-30 cm). It has to be mentioned that up to 18% of the cases required supplementary tacks besides the glue. A noteworthy mention is that the surgical intervention will need a particular type of mesh to adhere to this glue. This option seems promising, but extensive prospective randomized studies are required to compare the pain threshold, recurrence rate, and time to work between glue, tacks, and sutures.

**Figure 2:**
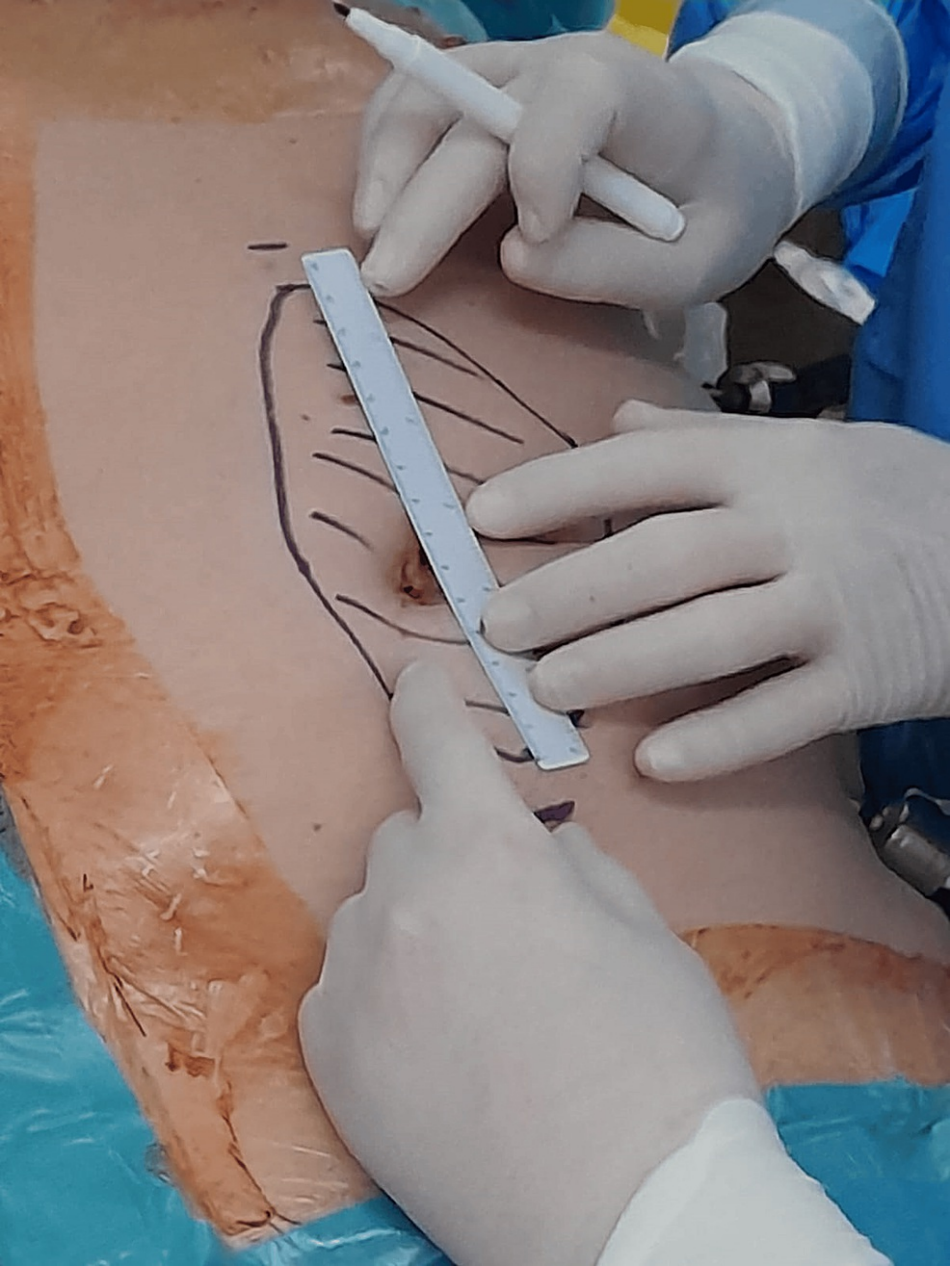
IPOMp for an umbilical hernia The shaded area represents where the mesh will be placed. Using a ruler, measure the distance from the hernia to the edge of the mesh. Observe the points superior and inferior to the shaded area where the anchor sutures will be placed. These sutures should be located 2-3 cm superior and inferior to the edge of the mesh to provide a bit of tension during ligation.

Regarding the mesh size, the current guidelines recommend IPOM for defects of up to 10 cm [4,40]. Before selecting the size, the defect must be measured. This can be done either by placing spinal needles through the circumference of the defect under laparoscopic control or in the intraperitoneal cavity using a sterile ruler. One can also measure the defect on the skin if it is an umbilical hernia, clearly visible, as demonstrated in Figure 2.

This technique needs to take into account the circumference of the abdomen, which is increased by the pneumoperitoneum. One should decrease the pressure to 7-8 mmHg before measuring. After the defect is measured, the convention is to have the margins of the mesh overlap the defect up to 5 cm circumferentially.

## Conclusions

Laparoscopic IPOM is a better option for IVH up to 10 cm than the open technique with regard to aesthetics, length of hospital stay, and postoperative pain. There is no difference in recurrence rates. Suturing the defect is beneficial, as it decreases seroma formation and maintains the functionality of the abdominal wall. Ideally, the suture should be executed intraperitoneally and laparoscopically. Regarding pain in mesh fixation, there seems to be an increase in the short term for the groups in which the mesh was anchored with sutures, but at six months, when compared to the tacks groups, there is no difference. New methods are being developed that include different types of glue to anchor the mesh but require large prospective randomized trials if they are to be included in the guidelines.
